# Comprehensive Assessment of Technological Challenges In Photovoltaic Waste Recovery In India Using Principal Component Analysis and Analytic Hierarchy Process Models

**DOI:** 10.1002/gch2.202400300

**Published:** 2025-02-24

**Authors:** Dinesh Yadav, Sanjeev Kumar, Prabhu Paramasivam, Praveen Kumar Kanti, Rupesh Gupta, Mohamed Yusuf

**Affiliations:** ^1^ Mechanical Engineering Department Delhi Skill and Entrepreneurship University Delhi 110077 India; ^2^ Department of Research and Innovation Saveetha School of Engineering, SIMATS Chennai Tamil Nadu 602105 India; ^3^ University Center for Research & Development (UCRD) Chandigarh University Mohali Punjab 140413 India; ^4^ Chitkara University Institute of Engineering and Technology Chitkara University Punjab 140401 India; ^5^ Department of Peace and Development Studies Njala University Bo Campus –18 Bo City 18 Sierra Leone

**Keywords:** AHP, PCA, PV waste, solar energy, technological challenges

## Abstract

The rapid expansion of photovoltaic (PV) technology has raised concerns about sustainable PV waste management, particularly in India, where inadequate infrastructure and technical limitations hinder effective recycling. Addressing these challenges is crucial for minimizing environmental risks and promoting a circular economy in the renewable energy sector. This study presents a smart multi‐criteria decision‐making (MCDM) approach that integrates Principal Component Analysis (PCA) and the Analytic Hierarchy Process (AHP) to assess technological challenges in PV waste management. PCA is applied to prioritize key challenges, while AHP evaluated their interrelationships through criteria weights. Despite the effectiveness of PCA and AHP, their combined application in PV waste recovery remains underexplored, particularly in the Indian context. Eight key challenges are identified, with hazardous recycling methods (83.2%) and low recycling potential (83.4%) ranking highest in PCA. AHP results highlighted the lack of advanced recycling technology (0.2298) and hazardous recycling methods (0.2084) as the most critical barriers. A multi‐criteria utility function is developed to illustrate these interdependencies. This research bridges critical knowledge gaps by offering data‐driven insights into PV waste recovery in India, contributing to sustainable waste management strategies and the development of an efficient recycling framework.

## Graphical abstract

Comprehensive investigations on PV waste recovery pathways in India. AHP identified that lack of advanced recycling technology is the biggest challenge. PCA identified that hazardous recycling methods (83.2%) is the main challenge.

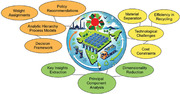

## 1 Introduction

Solar Photovoltaic (PV) contributes 3.6% of the world's electricity production and is still the third‐largest renewable electricity technology after hydropower and wind.^[^ ^1^, ^2^ ^]^ Solar panels are used to produce electricity from solar energy. The manufacturing and installation of solar panels started in 1990^[^ ^3^ ^]^ Solar energy is green and clean.^[^ ^4^, ^5^ ^]^ Currently, PV modules are being preferred for green and the lowest‐cost electricity generation in most of the world.^[^ ^6^ ^]^ Solar PV modules directly convert solar energy into electricity.^[^ ^7^, ^8^ ^]^

As per the guidelines of the International Technology Roadmap for Photovoltaics,^[^ ^9^ ^]^ the installed PV power capacity reached 500 GW in the year 2016 and is projected to attain 4500 GW by 2050. The average life of a solar panel is approximately 25 to 30 years.^[^ ^10^ ^]^ A 2016 report issued by the International Energy Agency Photovoltaic Power Systems Programme (IEA PVPS) and the International Renewable Energy Agency (IRENA) projected that global PV waste will reach 1.7–8 million tonnes by 2030 and that it will increase to 60–78 million tonnes by 2050.^[^ ^11^ ^]^ The International Renewable Energy Agency projects that the worldwide value of recoverable raw materials through end‐of‐life panels will amount to around $450 million by 2030, which is comparable to the price of the raw materials needed to create almost 60 million new panels. Installation of such a large number of solar panels and recycling infrastructure for PV waste in the future are required.^[^ ^12^, ^13^ ^]^ China, Germany, and Japan are predicted to produce the most cumulative EOL solar PV waste by 2030, and by 2050, the U.S. will surpass Germany, followed by Japan and India.^[^ ^10^ ^]^

Owing to the presence of several heavy metals, like tin and lead, in PV modules, serious environmental contamination problems may arise in the absence of proper recycling.^[^ ^14^ ^]^ Through the recycling process, it is possible to recover valuable metals such as silver, copper, and cadmium.^[^ ^3^, ^15^ ^]^ Therefore, worldwide, approaches to recycling solar panels are being created to lower the negative environmental effects of obsolete modules and to recover some value from solar PV waste. Presently, only Europe has effective regulations to gain economic benefits from recycling PV waste, but other countries have no specific regulatory framework to handle PV waste.^[^ ^16^ ^]^ At present, the European Union (EU) is the sole region with stringent regulations governing the recycling of photovoltaic (PV) waste, whereas other nations are in the process of formulating their systems. The EU's Waste Electrical and Electronic Equipment (WEEE) Directive compels PV panel producers to bear the costs of collecting and recycling their products. This directive also requires manufacturers to establish infrastructure for the free collection of waste from residential households. Various countries are at different stages of developing their own PV waste management systems, with some yet to implement regulations.^[^ ^17^ ^]^ For instance, the United States lacks federal‐level regulations for PV waste management, resulting in state‐by‐state variations in the requirements.

Countries, such as the USA, Japan, China, Australia, and India, are fast‐expanding solar markets without specific regulations for end‐of‐life (EOL) solar PV modules. These countries treat PV waste under a general regulatory framework for hazardous and non‐hazardous solid waste or WEEE.^[^ ^6^, ^18^ ^]^ PV waste is still considered unimportant compared to other electrical and electronic equipment waste, hence, currently setting up a plant for recycling PV waste is not economical.^[^ ^19^ ^]^ The PV module at the EOL is either dumped in landfills directly or recycled as per regulations to retrieve crucial material for reuse.^[^ ^20^, ^21^ ^]^ Recycling PV waste is essential for preventing environmental pollution and depleting the earth's mineral resources.^[^ ^22^ ^]^

Currently, PV waste handling follows an open loop cycle, so the raw material for PV module manufacturing mainly depends upon natural resources.^[^ ^23^ ^]^ The recycling process provides an opportunity to convert the open loop cycle to a circular loop cycle in the life cycle of the PV module.^[^ ^24^ ^]^ Solar panels have a high potential for the recyclability of PV materials.^[^ ^4^ ^]^ The absence of mandatory recycling regulations is the main barrier to accepting the recycling process.^[^ ^11^ ^]^ The lack of research on recycling technologies for PV waste handling is a major obstacle faced by the recycling sector.^[^ ^6^ ^]^

Different disposal and recycling methods and their impacts on the economy have been evaluated by Kong et al.^[^ ^25^ ^]^ Solar panel recycling method‐related research is occurring mainly in Europe, Japan, and the USA.^[^ ^26^ ^]^ Generally, the main focus of this research is focused mainly on the extraction of silicon and rare metals.^[^ ^27^ ^]^ For all PV technologies, the recycling process is still in the initial stages. The first recycling process for CdTe was developed by the USA‐based PV module manufacturer “First Solar”.^[^ ^28^ ^]^ The recycling process to recover valuable materials is based on the type of solar panels because each type of panel has a unique composition and differs from other types.

### 1.1 Photovoltaic Technologies

PV technologies are built on a variety of solar module types with structures that vary in size, weight, performance, and material composition. First‐generation, c‐Si‐based, PV technologies dominate the industry, accounting for 92% of all PV installations, i.e., 45% monocrystalline silicon panels and 55% multi‐crystalline silicon panels.^[^ ^1^ ^]^ Seven percent of the market is occupied by second‐generation thin‐film technologies, i.e., 5% CdTe and 2% Copper indium gallium selenide (CIGS), as listed in **Table** **1**. a‐Si panels have been abandoned because of their poor efficiency. The development of third‐generation modules, which primarily rely on technologies such as dye‐sensitized and organic PV cells, has only a 1% market share. Up to 2030, c‐Si panels are expected to continue to dominate the market. In comparison to CdTe, which is not anticipated to grow in market share, CIGS has the potential to increase efficiency.^[^ ^29^ ^]^ The characterization of the material and PV waste, as well as the economics of the treatment processes, will be influenced by the many components of major PV panel technologies. Thin‐film and silicon‐based panel designs have different construction methods, which are influenced by the manufacturing process. This study did not analyze novel or upcoming PV technologies because they are still in their research and development stages.

**Table 1:** Technological share of PV panels (2010–2030).

Technology	Panel	2010	2014	2020	2030
Silicon based	Mono‐crystalline Ribbon Multicrystalline a‐Si (amorph/micrograph)	86%	92%	73.3%	44.8%
Based on Thin‐film	CIGS Cadmium telluride (CdTe)	1% 13%	2% 5%	5.21% 5.2	6.4% 4.72%
Other types	Concentrating PV Organic PV Advanced c‐Si Heavy metals, alternatives of CIGS, advanced III‐V		1%	1.2% 5.79 8.7% 0.6%	0.58% 8.6 25.6% 9.4%

#### 1.1.1 Crystalline Silicon Panels

**Figure 1:**
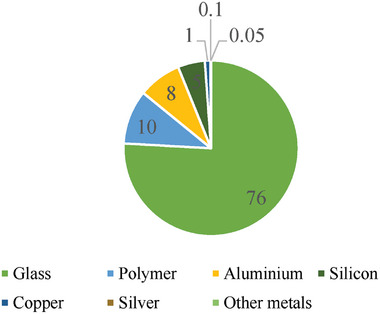
c‐Si module's composition.

c‐Si technology is created from solar‐grade silicon slices (wafers). Its components include an aluminum frame, glass, ethylene‐vinyl acetate (EVA), silicon cells, metallic connectors (copper, silver, and lead), and, in most cases, a polymer back sheet made of tedlar or polyethylene terephthalate (PET).^[^ ^30^ ^]^ Currently, a typical c‐Si PV panel weighs approximately 76% glass (the panel surface), 10% polymer (the encapsulant and the back‐sheet foil), 8% aluminum (frame), 5% silicon (the solar cells), 1% copper (the interconnectors), less than 0.1% silver (the contact lines), and other metals (mostly tin and lead).^[^ ^3^ ^]^ Silicon is the most crucial component of PV modules and accounts for approximately half of the module cost.^[^ ^22^ ^]^ The content of the glass in the c‐Si panels will be 4%–80% based on weight up to 2030. The largest material savings will come from a decrease in silicon from 5% to 3%, a decrease in aluminum of 1%, and a very modest decrease of 0.01% in other materials.^[^ ^9^ ^]^ The c‐Si module's composition is shown in **Figure** **1**. The market share of a‐Si PV panels has decreased significantly recently, and these panels do not comprise considerable amounts of valuable or dangerous components. They will probably not need specific waste treatment in the future.

#### 1.1.2 Thin Film Panels

Thin‐film‐type PV modules are low‐cost and contain very small quantities of materials. CIGS, CIGS, and a‐Si technologies are used for thin‐film solar panels.^[^ ^3^ ^]^ In terms of technology, thin‐film PV panels are more advanced than silicon‐based PV panels. By 2030, the glass content of c‐Si panels will likely increase.^[^ ^31^ ^]^ Less than 10% of the photovoltaic sector uses thin films.^[^ ^32^ ^]^ Currently holding 65%, 25%, and 10%, respectively, are amorphous silicon (a‐Si), copper indium gallium selenide (CIGS), as well as cadmium telluride (CdTe). The direct bandgap material of CIGS PV modules causes great light absorption capacity. Changing the ratios of the several elements of the compound semiconductor—e.g., indium, gallium, and selenium allows one to control the light spectrum.

**Figure 2:**
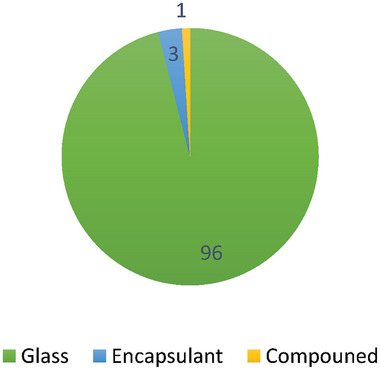
CdTe thin film module's composition.

The thin‐film technique that is most frequently utilized is CdTe. It contains a substantial amount of cadmium (Cd), a highly hazardous metal that poses an issue for the environment, as shown in **Figure** **2**.^[^ ^33^ ^]^ In comparison to other thin‐film technologies, a‐Si is less efficient, has lower durability, and is less hazardous. According to current predictions, the a‐Si module market will soon disappear since it cannot compete on price or efficiency.

Newer and latest technology‐based solar panels, i.e., organic cells, hybrid solar cells, and dye‐sensitized solar cells, are in their initial stages. New kinds of recycling procedures will be needed as a result of emerging technology. It is expected that the adverse effects on the environment due to harmful elements can be mitigated by using these latest solar panels. The mass installation of solar panels will soon generate a substantial portion of PV waste in India. There are many hurdles associated with managing such a massive volume of PV waste. To overcome these hurdles, technology will play a crucial role. The challenges that are overcome through the use of technology are described here as technological challenges or barriers.

This work is novel in its application of the integrated strategy of analytical hierarchical process (AHP) methodologies and principal component analysis (PCA) techniques to methodically separate the technological obstacles of PV waste management in India. Regarding environmentally friendly waste disposal for solar energy systems, this study offers a unique and comprehensive perspective by defining and rating important criteria, therefore opening the path for concentrated and effective solutions.

This study provides comprehensive knowledge of the major technological issues related to photovoltaic waste in India, which substantially contributes to previous studies. This study will have an impact on the future by influencing and directing further studies and legislative efforts, which will make it easier to create focused solutions for the sustainable management of PV waste.

## 2 Literature Review

Granata et al.^[^ ^34^ ^]^ proposed an integrated method that is viable for different PV technologies. This proposed integrated process route for the physical and chemical recycling treatment of both CdTe as well as Si‐based panels has a recycling rate of 91%. Furthermore, a better method for mechanically treating several types of photovoltaic panels was proposed. Lunardi et al.^[^ ^3^ ^]^ presented an overview of solar PV module recycling processes for both c‐Si and thin‐film technologies. Furthermore, the existing motivational laws, regulations, and recycling process were discussed, and possible issues were addressed in detail. It is difficult to retrieve valuable and toxic materials from PV modules through a recycling process, but this process has an amazing advantage in terms of environmental issues. The better utilization of technologies can generate a profitable outcome from the recycling sector.

Xu et al.^[^ ^27^ ^]^ studied various published research papers on the recycling of PV panels on a quantitative basis and suggested future directions for public policymakers. They also focused on technological research aspects that face many problems, and they further suggested developing an economically feasible and nontoxic recycling technology. Mahmoudi et al.^[^ ^35^ ^]^ reported that many countries still have not attempted to forecast PV waste and develop a recycling infrastructure. In further investigations, it was found that treatment processes, i.e., recycling, recovery, reuse, and landfill disposal, are among the top priorities of researchers. The development of an eco‐friendly recycling technology, such as reducing gas emissions and heat during various recycling processes, is a critical challenge.

Sica et al.^[^ ^29^ ^]^ studied and analyzed several research studies using a conceptual framework to characterize the primary technological and environmental effects of managing PV waste. Furthermore, they highlighted crucial components, prospective opportunities, and constraints emanating from technological, managerial, and organizational aspects to improve recycling as well as recovery rates. Nain and Kumar^[^ ^36^ ^]^ reported that regulations, incentives, and recycling infrastructure are major future challenges for the management of PV waste. Furthermore, they also found that 80% of respondents did not have any updates for their installed PV system after their EOL, 76% of producers or manufacturers did not recycle any type of PV module at their EOL, but 84% were interested in subsidized programs for recycling.

Chowdhury et al.^[^ ^37^ ^]^ focused on the present status of PV waste recycling, recycling technology, environmental protection, waste management, recycling policies, and the economic feasibility of recycling. It was also recommended to frame a better recycling policy and use cutting‐edge recycling technology in the future. Oteng et al.^[^ ^6^ ^]^ performed a scientific review of “trends in solar photovoltaic waste management research” and found that little attention was given to recycling, recovery, regulation, and policies on solar PV module waste management. Further suggestions were given to research current PV waste recycling practices, maximize recycling potential, and reduce hazardous emissions.

Balaji and Rahul^[^ ^38^ ^]^ proposed that proper policy and regulation for recycling PV waste at the EOL will create a sustainable and economical industry. The government and private organizations could establish a PPP model for recycling infrastructure to retrieve valuable and hazardous material. This approach was further recommended for circular economies and related challenges. Reich et al.^[^ ^22^ ^]^ investigated the experimental conditions to delaminate and recover silicon in the recycling process utilizing a mix of mechanical, thermal, and chemical approaches.

Jain et al.^[^ ^18^ ^]^ presented the current status of the PV waste management field and identified obstacles that prevent the effective recycling of PV waste at its end of life. Furthermore, a special regulatory framework has been constructed using a multistakeholder, multisectoral, and methodical approach. To mainstream PV waste management and the extended producer responsibility (ERP) approach. Patrick and Isherwood^[^ ^39^ ^]^ outlined the main solar panel recycling techniques for both crystalline silicon modules and thin‐film CdTe and CIGS modules. Rathore and Panwar^[^ ^40^ ^]^ mentioned that cutting‐edge research in the solar sector concentrates primarily on growing output without considering PV waste management and its impact on the environment and economy. Furthermore, they discussed the life cycle assessment of recycling technologies and technological development to recycle PV waste. Ganesan and Valderrama^[^ ^41^ ^]^ formulated an a‐LCA framework for the EOL management of c‐Si PV panels with the active participation of stakeholders and concluded that high‐value material recovery was the most justifiable option for PV waste management.

Iakovou et al.^[^ ^42^ ^]^ identified several significant challenges and restrictions, including the absence of helpful regulations and the absence of well‐structured and effective recycling infrastructure. They projected that establishing a sustainable reverse supply chain network for solar panels requires ongoing efforts to improve end‐to‐end supply chain costs, supported by empowering policies. Furthermore, they emphasized the importance of developing systematic decision‐making modeling outlines to explore various potentials and scenarios for optimizing solar waste reverse supply chain networks at the local, regional, and national levels. Lastly, they explored the development of a Resource Task Network (RTN)‐based model and established its practical applications and advantages through a case study.

Mathur et al.^[^ ^43^ ^]^ proposed an extension of industrial symbiosis for the circularization of the photovoltaic sector in the form of life cycle symbiosis. Through the establishment of cooperative business/economic contacts among a varied group of companies, we have implemented LCS in the context of EoL PVs by spotting waste streams that can have value as possible raw material/feedstock. While the water savings and power savings were 37735.65 m3 and 3615.11 MJ per metric ton of EoL PVs respectively, the averted global warming potential (GWP) and ecotoxicity were 2750 kg CO2 eq and 32000 CTUe respectively. In another study by Mathur et al.^[^ ^44^ ^]^ a system dynamics tool motivated by the materials recovery hierarchy to grasp the sustainability effects of EoU recovery was proposed. The model assesses environmental trade‐offs for alternative product recovery techniques giving top priority to the recovery of EoU goods, sub‐assemblies, components, and materials over more damaging alternatives of landfilling and incineration. The model is validated using a case study focusing on EoU solar photovoltaic (PV) panels.

An extensive analysis of the scholarly literature reveals a notable gap in the application of integrated MCDM frameworks. Although PCA and AHP are widely acknowledged as powerful analytical tools, their combined utilization in evaluating PV waste recovery pathways, especially within the Indian context, remains a largely untapped area of study. This research void presents an opportunity to exploit the synergistic potential of PCA and AHP for a more comprehensive assessment of PV waste‐recovery strategies in India. Integrating these methodologies could enable decision‐makers to acquire a deeper understanding of the intricate factors shaping PV waste management choices. Such an approach has the potential to yield a more nuanced perspective on the economic, environmental, and social dimensions of PV waste recovery in India, ultimately guiding more informed and sustainable policy formulation.

The innovative aspect of this study lies in its integration of AHP methodologies with PCA techniques to systematically address technological challenges in PV waste management in India. By identifying and prioritizing key criteria for the environmentally sustainable disposal of solar energy systems, this study presents a novel and comprehensive approach that facilitates targeted and efficacious solutions in the domain of photovoltaic waste handling.

This study fills critical knowledge gaps, offering crucial insights into the most efficient and long‐lasting methods for recovering PV waste in India. These findings contribute to the advancement of a more sustainable and circular solar energy sector.

## 3 Research Methodology

An integrated multicriteria decision‐making (MCDM) approach is employed for the analysis of technological challenges associated with PV waste management. These barriers are identified through a literature review, a questionnaire survey, and expert opinions. Integrated PCA and AHP approaches are used to examine primary data collected through questionnaires.

### 3.1 Principal Component Analysis (PCA)

The objective of PCA is to identify a reduced set of uncorrelated variables, termed principal components, that account for the majority of the variance in the initial data. Determined the optimal number of principal components retained using various criteria, such as 1eigenvalues and retaining components with eigenvalues exceeding 1. Scree plot analysis, conduct a visual assessment of the scree plot to identify the “elbow” where the curve's gradient begins to plateau. Subsequently, the variable loadings on each principal component. This examination facilitates the interpretation of the underlying factors or dimensions represented by individual components.

### 3.2 Analytic Hierarchy Process (AHP)

The AHP approach included the following steps: i). Established the primary goal at the highest level. ii). Decomposed the goal into various criteria iii). Divided criteria further into sub‐criteria. iv). Integrated identified barriers at the bottom level. v. Conducted pairwise comparisons between elements at each hierarchical level utilizing a rating system (Saaty's 1–9 scale) to indicate relative importance. vi). Obtained assessments from experts. vii). Evaluated the consistency of assessments using the Consistency Index (CI) and Consistency Ratio (CR). viii). Revised assessments to improve consistency if the CR exceeds an acceptable threshold. ix). Determined weights for each barrier based on the pairwise comparison results. x). Calculated the overall barrier priorities by aggregating weights across all hierarchical levels.

### 3.3 Integration of PCA and AHP

Finally, the principal components as criteria or sub‐criteria in the AHP hierarchy. This helped to cluster similar barriers and simplify the decision‐making process. This combined methodology enhances the decision‐making process by providing a more comprehensive and empirically‐based system for evaluating barriers. Through the integration of both approaches, researchers and practitioners can acquire a more nuanced comprehension of the complex interrelationships between obstacles and their relative importance. Furthermore, this integrated approach may prove particularly efficacious in domains necessitating the concurrent assessment of multiple, interrelated factors.

### 3.4 Ethical Approval Statements

a) The authors state that this study did not involve human participants as subjects. Instead, the study utilized a survey with experts in the field of solar energy materials to gather information. No medical testing or assessments were conducted, and the research does not involve human clinical data. Therefore, the study does not fall under the purview of the Declaration of Helsinki.

b) The written consent of the experts (human) was obtained for publishing this study as a research Paper.

## 4 Technological Challenges

The economical and eco‐friendly management of PV waste at the EOL is a major challenge for society. To handle these challenges, technology will play a crucial role, but there are some challenges against the use of technology to handle PV waste. The identified technological challenges are described in detail as follows:

### 4.1 Hazardous Method of Recycling (B1)

Currently, advancements in PV technology have focused mainly on the effectiveness of solar panels, without considering how they affect the environment after their EOL. There are currently no specific regulations or recommendations for managing PV waste in India hence, PV waste is recycled in a similar way as general e‐waste.^[^ ^10^ ^]^ PV waste contains heavy and hazardous metals that are harmful to human health and the environment.^[^ ^45^ ^]^ Effective and eco‐friendly methods for recycling PV waste have never been considered in India.^[^ ^40^ ^]^ There is an urgent need to manage the eco‐friendly disposal of solar panel waste.^[^ ^46^ ^]^

### 4.2 Inadequate Technical Skill Sets (B2)

Turaga et al.^[^ ^45^ ^]^ examined that a lack of technical skill sets is a major issue in managing PV waste in India. Prakash et al.^[^ ^47^ ^]^ also stated that the “lack of technical skill sets” is a major issue in the management of PV waste in India. Vrat et al.,^[^ ^10^ ^]^ in the analysis of e‐waste management in India, mentioned that a lack of technical skill sets is crucial for sustainable management. Chowdhury et al.^[^ ^37^ ^]^ suggested the use of fully equipped technical skill sets for sustainable PV waste disposal management.

### 4.3 Unavailability of the Latest Technology for Recycling (B3)

PV waste is dissimilar from e‐waste; hence, recycling technology for general e‐waste is not suitable for recycling PV waste.^[^ ^10^ ^]^ Unfortunately, in India, no specific policy or guidelines are available. A major portion of e‐waste and PV waste is collected and recycled by informal recyclers with older and traditional technology, without knowing its impact on the environment.^[^ ^46^ ^]^ Furthermore, the recovery rate of materials through older recycling technology is much lower.^[^ ^40^ ^]^ The unavailability of the latest technology is a major technological challenge for handling PV waste in India.

### 4.4 High Cost of Sustainable Technology (B4)

Sustainable technology has no adverse effect on the environment.^[^ ^48^ ^]^ In India, the major recycling sector for PV waste is the informal sector, which has older technology.^[^ ^46^ ^]^ Due to the high cost of the latest sustainable technology, the informal sector cannot afford it. The government has no financial support to restructure the older recycling system with sustainable technology.^[^ ^49^ ^]^ Due to the high cost of sustainable technology, PV waste management in India is tedious.^[^ ^47^ ^]^

### 4.5 Poor Technological Infrastructure (B5)

Technological infrastructure refers to a material recovery process, maintenance and repair, a recycling process, and a PV waste handling technique.^[^ ^50^ ^]^ Poor technical infrastructure is a major hurdle for efficient PV waste management in India.^[^ ^46^ ^]^ Advancements in technological infrastructure are needed to handle massive PV waste generation in the future.

### 4.6 Lack of Technological Monitoring for the Return of PV Waste (B6)

The lack of technology that can be used to monitor the path of flow to end‐of‐life disposal of PV waste is a major challenge in India. Due to this lack of awareness, the collection of PV waste in India is tedious.^[^ ^10^ ^]^ Vrat et al.^[^ ^46^ ^]^ found that the lack of technological monitoring for the return of PV waste is a crucial challenge. This barrier is also defined by Prakash et al.^[^ ^47^ ^]^ With the help of information technology, an effective online monitoring system can be created.

### 4.7 Technologically Unskilled Manpower (B7)

Manpower is the backbone of any system for smooth functioning.^[^ ^37^ ^]^ Upgradation of technical knowledge is necessary for manpower to handle and perform efficiently.^[^ ^46^ ^]^ Currently, technically skilled manpower is needed to handle PV waste in India.

### 4.8 Low Recycling Potential (B8)

The best way to sustainably manage PV waste is through recycling. The final goal of any waste management system is to recover the maximum amount of material from waste without any adverse effects on the environment.^[^ ^51^ ^]^ Due to its low recycling capacity, a large amount of PV waste is disposed of in landfills without recycling.^[^ ^37^ ^]^ This has led to considerable economic losses and damage to environmental conditions.

All the technological challenges associated with PV waste management are listed in **Table** **2**.

**Table 2:** Technological challenges of PV waste management in India.

Symbol	Technological challenges	References
B1	Hazardous method of recycling.	Neelam & Panwar^[^ ^40^ ^]^; Gautam et al.^[^ ^10^ ^]^; Turaga et al.^[^ ^45^ ^]^; Vrat et al.^[^ ^46^ ^]^; Prakash et al.^[^ ^47^ ^]^
B2	Inadequate technical skill sets.	Khan et al.^[^ ^51^ ^]^; Chowdhury et al^[^ ^37^ ^]^; Vrat et al.^[^ ^46^ ^]^; Prakash et al.^[^ ^47^ ^]^
B3	Unavailability of the latest technology of recycling.	Neelam & Panwar^[^ ^40^ ^]^; Gautam et al.^[^ ^10^ ^]^; Turaga et al.^[^ ^45^ ^]^; Vrat et al.^[^ ^46^ ^]^; Prakash et al.^[^ ^47^ ^]^
B4	High cost of sustainable technology.	“Franchina et al.^[^ ^48^ ^]^; Turaga et al.^[^ ^45^ ^]^; Vrat et al.^[^ ^46^ ^]^; Prakash et al.^[^ ^47^ ^]^
B5	Poor technological infrastructure.	Neelam & Panwar^[^ ^40^ ^]^; Chowdhury et al^[^ ^37^ ^]^; Turaga et al.^[^ ^45^ ^]^; Vrat et al.^[^ ^46^ ^]^; Martínez et al.^[^ ^50^ ^]^; Prakash et al.^[^ ^47^ ^]^
B6	Lack of technological monitoring to return PV waste.	Khan et al.^[^ ^51^ ^]^; Chowdhury et al^[^ ^37^ ^]^; Turaga et al.^[^ ^45^ ^]^; Vrat et al.^[^ ^46^ ^]^; Prakash et al.^[^ ^47^ ^]^
B7	Technologically unskilled manpower	Chowdhury et al^[^ ^37^ ^]^; Turaga et al.^[^ ^45^ ^]^; Vrat et al.^[^ ^46^ ^]^; Martínez et al.^[^ ^50^ ^]^; Prakash et al.^[^ ^47^ ^]^
B8	Low recycling potential	Neelam & Panwar^[^ ^40^ ^]^; Khan et al.^[^ ^51^ ^]^; Turaga et al.^[^ ^45^ ^]^; Vrat et al.^[^ ^46^ ^]^; Prakash et al.^[^ ^47^ ^]^

## 5 Analysis of Data

A quantitative research approach is used to conduct a questionnaire survey. The quantitative research method involves the collection of numerical data, which are further analyzed for various parameters.^[^ ^52^ ^]^ Here, a closed‐ended questionnaire is used to obtain information from experts. Google Forms were used to distribute the questionnaire among the target population. The questionnaire consisted of two sections. Information about the respondents was retrieved in the first part. The second portion compiles data on technological challenges. Two hundred participants were asked to select the best option from a questionnaire using a five‐point Likert scale to rate agreement or disagreement with barriers. For the statistical research, the target populations were researchers, professionals, academics, industrial technical staff, and technical colleges and university students. A total of 86 responses were obtained from the respondents and were considered suitable primary data for further analysis. The overall response rate was 43%, which is more than the minimum 20% response rate recommended by Malhotra and Grover.^[^ ^53^ ^]^ This analysis was performed by using PCA with the help of Statistical Package for Social Science (SPSS) software and the analytic hierarchy process (AHP).

### 5.1 PCA Analysis

**Figure 3:**
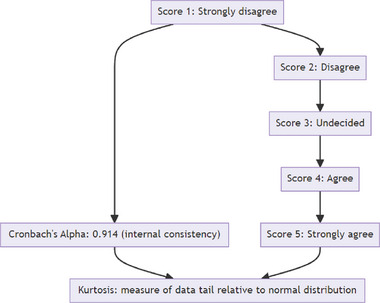
Likert scale.

**Figure 4:**
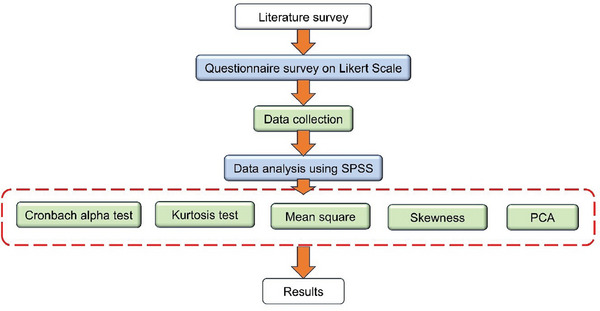
PCA Research Methodology.

PCA is a mathematical technique that uses a small number of parameters to define the variance in a dataset (i.e., the responses used to characterize the samples).^[^ ^54^ ^]^ Many researchers have utilized PCA in a variety of scientific fields and have made numerous adjustments to it before arriving at its current form since it is frequently thought to be a better modeling alternative for response data than analysis of variance.^[^ ^55^ ^]^ Principal component analysis (PCA) is used to reduce large data into clusters by exploring the fundamental theoretical structure of variables.^[^ ^56^ ^]^ The Likert scale measures opinions on a predesigned numerical value scale from 1 to 5, as shown in **Figure** **3**. Each number on the scale is associated with an opinion such as 1‐Strongly disagree, 2‐Disagree, 3‐Undecided, 4‐Agree, or 5‐Strongly agree. Variables with average values greater than 3.00 were considered for analysis.^[^ ^57^ ^]^ After the various experts responded to the questionnaire, the internal consistency (reliability) of the Likert scale was checked.^[^ ^58^ ^]^ The PCA research methodology is shown with a flow chart in **Figure** **4**. Reliability is defined as consistency in results after repeated measurements.^[^ ^59^ ^]^

From **Table** **3**, we can get Cronbach's Alpha Value. As shown in **Table** **4**, Kurtosis values are within the acceptable limits. Kurtosis values between ‐2 and 2 are considered acceptable to prove a normal univariate distribution.^[^ ^60^ ^]^ Skewness values between ‐1 and +1 were considered to indicate a normal distribution. The skewness values in Table 4 are within limits; hence, the distribution of items is normal.

**Table 3:** Reliability statistics.

Cronbach's Alpha	Cronbach's Alpha Based on Standardized Items	N of Items
0.914	0.914	8

**Table 4:** Descriptive statistics.

Variables	Minimum	Maximum	Mean	Std. Deviation	Skewness		Kurtosis	
	Statistic	Statistic	Statistic	Statistic	Statistic	Std. Error	Statistic	Std. Error
B1	1.00	5.00	3.6860	1.30395	−.892	.260	−.259	.514
B2	1.00	5.00	3.6744	1.26901	−.562	.260	−.861	.514
B3	1.00	5.00	3.5698	1.29742	−.604	.260	−.753	.514
B4	1.00	5.00	3.8140	1.22245	−.742	.260	−.419	.514
B5	1.00	5.00	3.8488	1.21285	−.878	.260	−.196	.514
B6	1.00	5.00	3.9535	1.23581	−.620	.260	−.042	.514
B7	1.00	5.00	3.7442	1.25732	−.700	.260	−.660	.514
B8	1.00	5.00	3.8023	1.21578	−.535	.260	−.327	.514

The Kaiser‒Meyer‒Olkin measure of sampling adequacy shows that the distribution of data is appropriate for PCA. From **Table** **5**, its value is 0.884, which is higher than the permitted threshold value of 0.6. Furthermore, Bartlett's test of sphericity yielded a result of 0.000, which is less than 0.05 (criteria limit), showing that variables have a statistically significant relationship.

**Table 5:** KMO and Bartlett's test.

Kaiser–Meyer–Olkin Measure of Sampling Adequacy.		0.884
Bartlett's Test of Sphericity	Approx. Chi‐Square	428.675
	Df	28
	Sig.	.000

**Figure 5:**
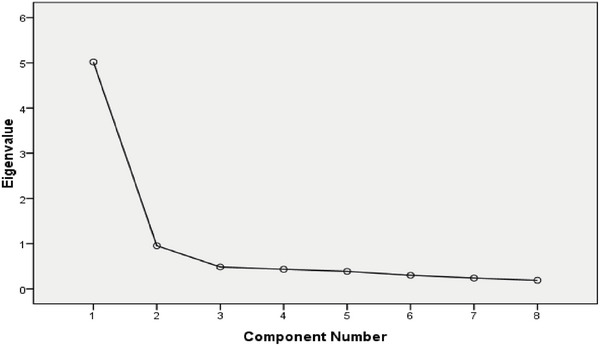
Scree plot for PCA.

**Figure 6:**
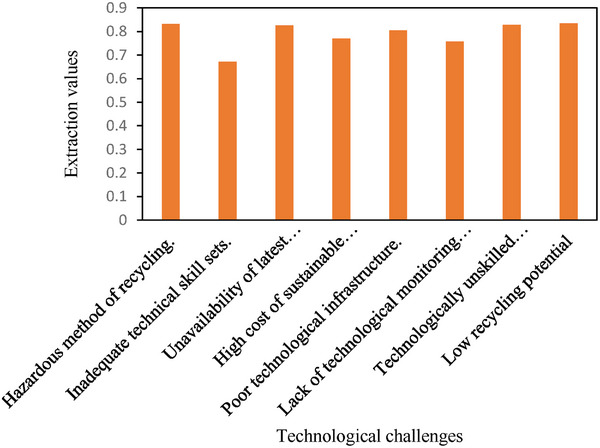
Graphical representation of component variables (Extraction method).

**Figure** **5** reveals the extreme slopes of the components, which caused them to break below an eigenvalue of 1.0 after 1 component to display that just one component satisfied the conditions set out by Kaiser's eigenvalues. According to the PCA results shown in **Table** **6**, only one component was extracted. A graphical representation of the values of the component variables is shown in **Figure** **6**. As shown in **Table** **7**, 62.735% of the total variables were cumulatively explained by this component.

**Table 6:** Component matrix (Extraction method: principal component analysis).

Variables	Extraction values
B1	.832
B2	.672
B3	.826
B4	.769
B5	.804
B6	.757
B7	.828
B8	.834

**Table 7:** Total variance explained.

Component	Total	% of variance	Cumulative %
1	5.019	62.735	62.735

The descriptive analysis results obtained by using PCA are presented in Table 6. The component matrix extracted through PCA is presented in Table 7, which shows that there is only one component loaded with eight technological challenges. The extracted values of the component variables show their impact on PV waste management through technology intervention. The values of the extracted technological challenges are as follows: hazardous method of recycling (0.832), inadequate technical skill sets (0.672), unavailability of the latest recycling technology (0.826), high cost of sustainable technology (0.769), poor technological infrastructure (0.804), lack of technological monitoring to return PV waste (0.757), technically unskilled manpower (0.828) and low recycling potential (0.834). A total cumulative variance of 62.735% of the entire variance of variables is evaluated by PCA.

### 5.2 AHP Analysis

Saaty^[^ ^61^, ^62^ ^]^ proposed an analytical hierarchical process (AHP) to arrive at weightings for the criteria based on the importance of the different criteria. The AHP approach was used to construct a hierarchy table of technological challenges. According to Saaty,^[^ ^63^, ^64^ ^]^ the AHP is a mathematical framework that uses consistent matrices and their corresponding eigenvectors to produce weights for complex decisions that can be either subjective or objective. Due to the inconsistency of human decisions, a modest amount of judgmental inconsistency is permitted. The eight finalized technological challenges were analyzed by using AHP.

There are five steps in the design of the AHP methodology framework. 1) Identify the issue and its objective or goal, 2) create a pairwise comparison matrix using the Saaty scale, as shown in **Table** **8**, 3) determine how to normalize the matrix and determine the weights for each variable, 4) verify the matrix's consistency ratio, which must be smaller than 0.1, and 5) determine the priority ranking if the matrix is consistent.

**Table 8:** Saaty (1980) scale of relative importance.

Preference weights/level of importance	Definition	Reciprocal
1	Equal importance	1
3	Moderate importance	1/3
5	Strong importance	1/5
7	Very strong importance	1/7
9	Extreme importance	1/9
2,4,6,8	Intermediate importance	1/2,1/4,1/6,1/8

The AHP analysis of technological challenges is described in the following steps:

**Step 1**: The priorities of technological challenges are the goal of this analysis.
**Step 2**: This stage determines the relative significance of certain characteristics or criteria concerning the goal. A pairwise comparison matrix was created by using the Saaty scale, as illustrated in **Table** **9**.

**Table 9:** Pairwise comparison matrix for technological challenges.

	B1	B2	B3	B4	B5	B6	B7	B8
B1	1	2	0.5	5	4	2	2	2
B2	0.5	1	0.5	2	2	1	2	2
B3	2	2	1	2	2	2	3	3
B4	0.2	0.5	0.5	1	0.5	0.5	0.5	0.5
B5	0.25	0.5	0.5	2	1	0.5	0.5	0.5
B6	0.5	1	0.5	2	2	1	2	2
B7	0.5	0.5	0.333	2	2	0.5	1	2
B8	0.5	0.5	0.333	2	2	0.5	0.5	1

**Step 3**: The criterion weights calculated from the pair‐wise matrix are shown in **Table** **10**.

**Table 10:** Criteria weights of technological challenges.

Technological Challenges	Criteria weights
B1	0.2084
B2	0.1287
B3	0.2298
B4	0.0564
B5	0.0686
B6	0.1287
B7	0.0972
B8	0.0822

**Step 4**: The consistency index for the matrix of order n is computed by the formula CI = (λ_max_− n)/(n‐1), and the consistency ratio is calculated by using the formula CR = CI/RI, where the RI value is taken from **Table** **11**. For n equal to 8, the RI value = 1.41.

**Table 11:** The random consistency index (Saaty, 1980).

n	1	2	3	4	5	6	7	8	9	10
Random Index (RI)	0	0	0.58	0.90	1.21	1.24	1.32	1.41	1.45	1.49

If CR<0.10, then the matrix is acceptable. Here, the calculated λ_max = _8.3908, CI = 0.05583, and CR value = 0.0396.

Since CR<0.10, hence pair‐wise comparison matrix of technological challenges shown in Table 11 is consistent.

**Figure 7:**
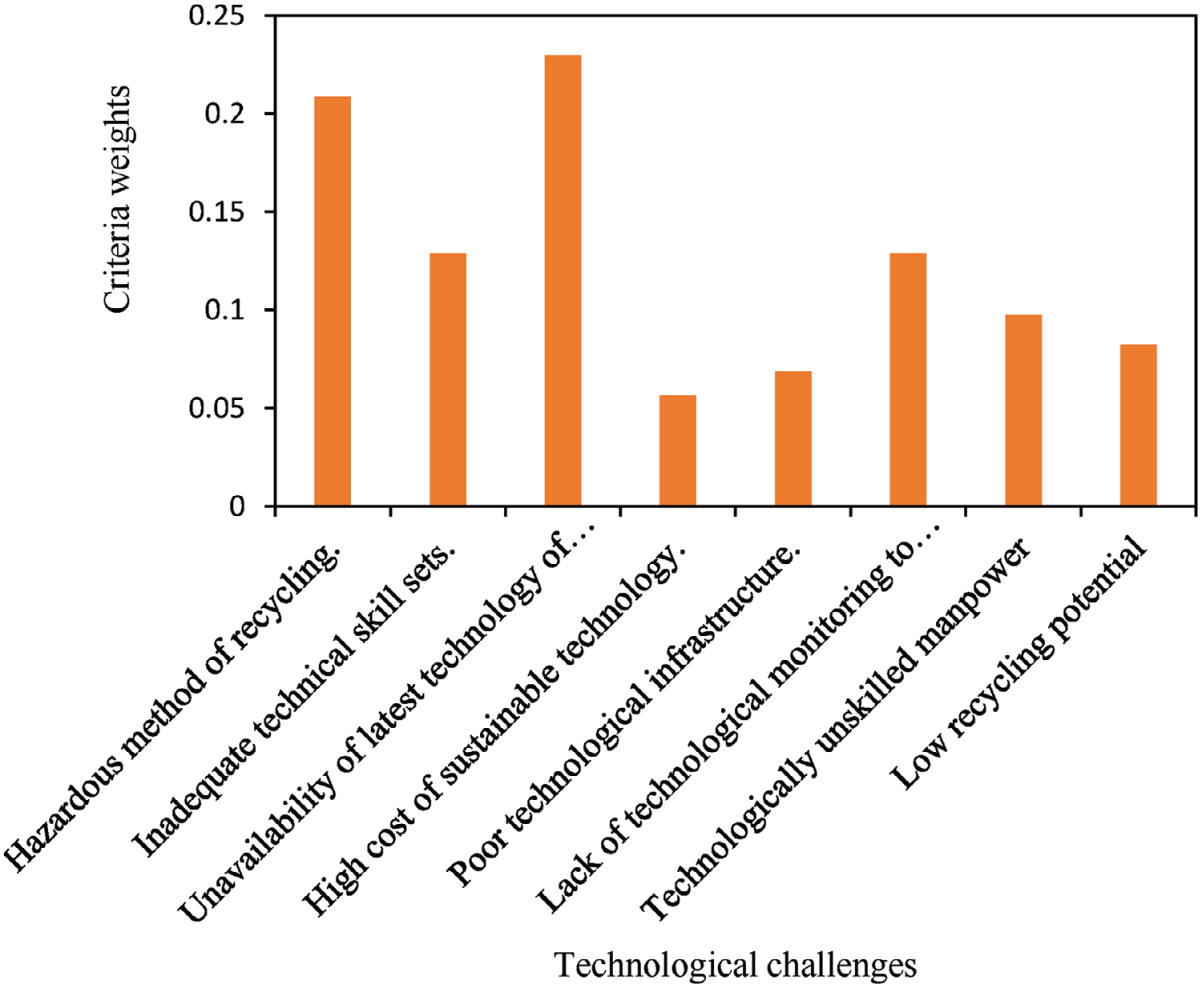
Graphical representation of the priorities of technological challenges.

**Step 5**: The AHP weights for each technological challenge are obtained, as shown in **Table** **12**, and the relative priorities are graphically represented in **Figure** **7**.

**Table 12:** Sorted weights and ranking of technological challenges.

Barrier	Sorted weights	Ranking
Hazardous method of recycling.	0.2084	2
Inadequate technical skill sets.	0.1287	3
Unavailability of latest technology of recycling.	0.2298	1
High cost of sustainable technology.	0.0564	7
Poor technological infrastructure.	0.0686	6
Lack of technological monitoring to return of PV waste.	0.1287	3
Technologically unskilled manpower	0.0972	4
Low recycling potential	0.0822	5

A multicriteria utility mathematical function (U) was retrieved to show the interrelationship among technological challenges as follows:

(1) $$\begin{array}{ccc} U & = & 0.21\times B1+0.13\times B2+0.23\times B3+0.06\times B4+0.07\times B5\\ & & +\;0.13\times B6+0.1\times B7+0.08\times B8 \end{array}$$

The criterion weights of each technological challenge were sorted out by determined via the AHP approach. A ranking was given to each factor on the basis of their criteria weights, and relative priorities were determined. Furthermore, a mathematical function showing interrelationships among challenges was proposed. Unavailability of the latest recycling technology, hazardous recycling methods, lack of technological monitoring for the return of PV waste, inadequate technical skill sets, and technologically unskilled manpower are the top five technological challenges, followed by low recycling potential, poor technological infrastructure and high cost of sustainable technology for PV waste management in the Indian context.

## 6 Discussions

The technological challenges associated with photovoltaic (PV) waste recovery were analyzed using two well‐established multi‐criteria decision‐making techniques: Principal Component Analysis (PCA) and the Analytic Hierarchy Process (AHP). These approaches provide a disciplined evaluation of the most important obstacles influencing the effectiveness and sustainability of PV waste management in India.

The most important technological issues found by the PCA analysis were limited recycling potential (0.834), dangerous ways of recycling (0.8832), and technologically untrained workers (0.828). These problems imply that the PV waste recovery industry in India suffers from inefficiencies in material recovery techniques, therefore maybe causing environmental damage and financial losses. The high rating of low recycling potential suggests that current techniques fail to efficiently recover valuable elements from end‐of‐life PV modules, perhaps due to obsolete technology and inadequate process optimization.

The dangerous recycling techniques (0.832) aggravate the issue even further as incorrect disposal and engagement in the informal sector could cause toxic emissions, groundwater pollution, and health hazards. The lack of workforce training and knowledge shown by technologically unskilled workers (0.828) directly affects the efficiency and safety of PV waste management. Other difficulties like inadequate technological infrastructure (0.804) and unavailability of the newest recycling technologies (0.826) point to the industry lacking the required developments in processing techniques and modern equipment. The efficiency of material recovery stays poor without access to current recycling technologies, which lowers the financial incentives for processing big PV waste. The great cost of sustainable technologies (0.769) is still a key obstacle as funding for modern equipment and recycling centers calls for large financial resources. The absence of government incentives or private sector investment restricts the acceptance of new technology even more, thereby making it challenging to increase PV waste recovery initiatives. Furthermore, lacking technology monitoring for PV waste return (0.757) suggests inefficiencies in identifying and managing the defunct PV modules, hence causing uncontrolled disposal and loss of precious resources. Though it adds to the more general problem of labor constraints, the lowest‐ranked challenge inappropriate technical skill sets 0.671 still has importance. Effective implementation of best practices in PV module deconstruction, material separation, and hazardous waste management depends on appropriate training and information transfer; without them, operators cannot with just one main component recovered from the PCA results which explains 62.735% of the total variance—these technological difficulties are presumably interrelated and help to explain the inefficiencies of PV waste recovery. This large variation % shows that the found problems are highly linked and need for combined policy and technical solutions to be solved.

By assigning criterion weights depending on expert judgments, the AHP approach was applied to quantify the relative relevance of every technical problem. The findings indicated that the most important obstacle is the lack of the most recent recycling technologies (0.2298), therefore stressing the immediate necessity of investment in improved material recovery technologies. PV waste recycling will remain ineffective without current processing tools, therefore increasing the environmental impact of disposed of modules. Reiterating the PCA results, the hazardous way of recycling (0.2084) came second in importance. Often using acid leaching and open‐air combustion, informal recycling methods carry great environmental and health hazards. Reducing these detrimental effects depends on established organized, controlled recycling processes. Given equal weight was the absence of technical monitoring for PV waste return (0.1287) and insufficient technical skill sets (0.1287), implying that tracking and workforce capabilities are critical elements in enhancing waste recovery efficiency. While trained personnel guarantees safe and efficient recycling, monitoring guarantees correct collecting and processing of PV waste. Received lesser but still substantial weights were other obstacles like technologically untrained workers (0.0972), limited recycling potential (0.082), weak technical infrastructure (0.0686), and high cost of sustainable technology (0.0564).

These results imply that, although they are secondary in importance, these elements are nonetheless quite important in deciding the general effectiveness of PV waste management. To organize the interdependencies among the technical difficulties found by PCA and AHP, a multi‐criteria utility function was devised. The results show a clear relationship between worker skills, hazardous recycling techniques, and the availability of recycling technologies. While untrained workers worsen waste processing inefficiencies, the lack of innovative technology aggravates hazardous recycling methods. The low acceptance of new recycling techniques is connected to the high cost of sustainable technology, which emphasizes the need for legislative interventions and financial incentives to help the creation of sophisticated PV waste recovery facilities. Furthermore, impeding the gathering of end‐of‐life PV modules and thus their appropriate disposal and recovery are inadequate technical monitoring systems. The results highlight the need for a multifarious strategy to solve the technological difficulties in PV waste management. Important advice includes:

- Investment in Advanced Recycling Technology: Modern PV module recycling facilities fitted with effective material recovery systems should have priority in government and commercial sector cooperation.
- Regulation of Informal Recycling Practices: Strict environmental rules and enforcement systems assist in eradicating dangerous recycling techniques and promote the safe processing of informally recycled products.
- Workforce Training and Capacity Building: Establishing skill development programs targeted at PV waste management and recycling processes can help to increase process efficiency and safety by employing workforce training and capacity building.
- Financial Incentives: Grants, tax exemptions, and subsidies for sustainable technologies serve to lower the financial load of funding advanced recycling infrastructure.
- Integration of PV Waste Monitoring Systems: To improve the efficiency of collecting garbage, digital tracking, and reporting systems have to be included in waste management strategies.

## 7 Conclusion 

PCA and AHP are used in tandem to identify and evaluate technological challenges related to PV waste management in India. The major findings are the extracted values of the component variables and the interrelationships among technological challenges. The major technological challenges limiting PV waste management are low recycling potential, hazardous recycling methods, and technologically unskilled manpower, with PCA values of 0.834, 0.832, and 0.828, respectively. Criteria weights of 0.2298, 0.2084, 0.1287, and 0.1287 are strongly correlated with the unavailability of the latest recycling technology, recycling methods, lack of technological monitoring for the return of PV waste, and inadequate technology.

Through the use of sustainable technology, this study helps to establish an eco‐friendly and effective PV waste management approach for the future. In the future, a sustainable resilience path for PV waste treatment may be framed using the ISM and DEMATEL decision‐making approaches. Another area of future research will be the identification and evaluation of technological enablers for PV waste management.

## Conflict of Interest

The authors declare no conflict of interest.

## Author Contributions

D.Y. performed conceptualization, data curation, investigation, methodology, supervision; project administration and wrote the final manuscript. S.K. performed validation, supervision and wrote the original draft. P.P. and P.K.K. performed software, methodology wrote reviewed and edited the final manuscript. R.G. and M.Y. performed formal analysis, methodology wrote reviewed and edited the final manuscript.

## Ethical Approval

There was no ethics or institutional committee in place at a researcher's institution at the time the study was conducted. The authors state that the study does not involve human participants.

## Data Availability

The data that support the findings of this study are available from the corresponding author upon reasonable request.
